# Challenges in Translating Regenerative Therapies for Spinal Cord Injury

**DOI:** 10.46292/sci23-00044S

**Published:** 2023-11-17

**Authors:** Andrew N. Stewart, John C. Gensel, Linda Jones, Karim Fouad

**Affiliations:** 1 Spinal Cord and Brain Injury Research Center, University of Kentucky, Lexington, Kentucky, USA;; 2 Department of Occupational Therapy, Thomas Jefferson University, Philadelphia, Pennsylvania, USA;; 3 Department of Physical Therapy, University of Alberta, Edmonton, Canada

**Keywords:** animal models, combined treatments, interspecies differences, publication transparency, translational success

## Abstract

Regenerating the injured spinal cord is a substantial challenge with many obstacles that need to be overcome to achieve robust functional benefits. This abundance of hurdles can partly explain the limited success when applying regenerative intervention treatments in animal models and/or people. In this article, we elaborate on a few of these obstacles, starting with the applicability of animal models and how they compare to the clinical setting. We then discuss the requirement for combinatorial interventions and the associated problems in experimental design, including the addition of rehabilitative training. The article expands on differences in lesion sizes and locations between humans and common animal models, and how this difference can determine the success or failure of an intervention. An additional and frequently overlooked problem in the translation of interventions that applies beyond the field of neuroregeneration is the reporting bias and the lack of transparency in reporting findings. New data mandates are tackling this problem and will eventually result in a more balanced view of the field. Finally, we will discuss strategies to negotiate the challenging course of successful translation to facilitate successful translation of regeneration promoting interventions.

## Introduction

Following injury of the central nervous system (brain and spinal cord), there is only very limited spontaneous repair, including cell replacement or regrowth of injured axons in rodents. The extent of repair in humans is unknown, but it is likely similarly restricted. There are also no potent interventions available to enable such repair, despite centuries of research, substantial investment, and many reported breakthroughs in animal models.^1^ The unsuccessful translation from the bench to the bedside raises the question about the usefulness of animal models in the translational pipeline to assess efficacy, a common question in the neurological sciences. However, we must acknowledge that interventions under development and tested to repair the injured spinal cord utilize animal models that only moderately mimic the clinical pathology. Further, while preclinical study design often deviates substantially from the clinical setting (including lesion characteristics and variability), clinical trials can also often deviate from the evidence provided by preclinical investigations in variables that may be essential for the efficacy of an intervention (lesion completeness, time postinjury, etc.). Furthermore, there are many other variables that challenge the translatability of findings from animal models, such as a publication bias toward positive results,^2^,^3^,^4^ the ability to examine multiple outcome measures in animal studies (vs. the typical requirement for a single primary endpoint in late stage clinical trials), the lack of blinding and randomization, and the limited reproducibility of findings that can be interpreted as more promising than they may in fact be.^5^ In this review, we will discuss challenges in the process of discovery research to translating regenerative treatments in the field of spinal cord injury (SCI), the value and progress of research in animal models, and strategies to successfully move forward.

## Challenges with Animal Modeling as the Preclinical Standard in SCI Translation

### Species differences: Do they matter?

Despite obvious differences between humans and other species, about 85% of genes are identical between rodents and humans.^6^,^7^ Nearly every gene found in one species has been found in a similar form in the other. Of the approximately 4000 genes that have been studied, less than 10 are found in one species but not in the other (genome.gov), and epigenetic differences are only slowly being explored.^8^ The genetic similarity among species suggests that many molecular underpinnings and responses to environmental stimuli are likely to be conserved among species. For example, in both humans and rodents, axons do not regenerate in the central nervous system and inhibitors for neurite outgrowth (e.g., Nogo A, myelin-associated glycoprotein [MAG], chondroitin sulfate proteoglycans [CSPGs]) are found in both species.^9^,^10^,^11^ The value of rodent models has been discussed elsewhere,^12^,^13^ pointing out various commonalties but also differences. Both humans and rodents show similar motor, sensory, and autonomic deficits after injuries, and rodents as well as humans suffer from a lack of axonal regeneration and cell replacement. However, despite many physiological, anatomical, and biochemical responses to SCI, some noteworthy differences exist, which have important implications for translatability. For example, humans and rats show similarities at the lesion site,^14^, ^15^,^16^ including the formation of cystic cavities and a fibrous scar surrounding the injury, whereas mice experience cells proliferation in the injury area and typically do not form fluid-filled cysts.^17^,^18^ Similarly, in both mice and rats the predominate location of descending corticospinal tract (CST) fibers is in the dorsal column, whereas in humans the CST descends primarily through the lateral columns.^19^,^20^ Other important species differences can be found in the neuroanatomy (e.g., the role/existence of the rubrospinal tract [RST])^21^ and the significant difference in physical size. Size differences (e.g., between a mouse and a human) can affect various aspects of repair and recovery. For example, a larger distance across which axons have to regenerate to reach a target will make an intervention more challenging. Another size-related example includes the challenge to maintain body temperature in a rodent (especially during surgery when an injury is applied), which also highlights metabolic differences among species with heart rates in mice that easily reach over 500 beats per minute. A generally faster metabolism in rodents potentially reduces the half-life of drugs and many other treatments including those designed to promote axonal regeneration.^22^

All the differences between rodents and humans challenge the interpretation of how recovery at a functional level in rodents translates to the human condition. Indeed, even outcomes such as locomotion, which has been extensively studied, are challenging to extrapolate between species. Despite obvious differences between quadrupeds (rodents) and bipeds (humans), it is essential to understand the mechanisms that induce locomotor recovery for translational relevance. The relationship between increasing spared tissue using neuroprotective strategies and improved locomotor function is both logical and well described, but the relationship between recovery of function from regenerative-based strategies is significantly more challenging to understand and to extrapolate to the human condition. However, despite translational challenges at the functional level, there is currently no evidence that at an anatomical/biochemical level, regeneration-promoting treatments will differ between species in the ability to promote repair.

Extrapolating findings from rodent models into humans presents challenges that are not always based directly on interspecies differences but rather on how animal models are applied. Different experimental injury models confer unique opportunities for recovery and therefore potentially different responses to treatments,^23^ which may not accurately capture the clinical condition. For example, emerging evidence suggests that astroglia bridges spanning the lesion in experimental crush models are essential for observing successful axon regeneration using gene therapy–based approaches to induce axon growth.^24^,^25^ Importantly, astroglia bridges do not form when lesions are too large, which, in the context of animal models, is a relatively small lesion approximating 0.5 to 1 mm rostro-caudal extent.^24^ Lesion sizes in humans will rarely, if ever, be 0.5 mm in thickness, but instead they can approximate several centimeters or more. A great deal can be learned from animal models in terms of biology and the conditions needed to observe axon regeneration in the spinal cord, but such findings do not necessarily directly translate the human condition.

Clinical SCI frequently differs from animal models: (1) Unlike rodent models, humans frequently suffer from comorbidities such as multiorgan trauma.^26^ (2) Anesthetics used during injury procedure in rodents are not as well controlled, with pulse oxygenation frequently dropping below 90%. (3) Different injury biomechanics in humans, such as a contusion, are frequently combined with compression. (4) Injuries in humans span larger rostro-caudal areas compared to rodents that are usually limited to one spinal segment. (5) There is more variability in impact velocities in humans (higher in humans).^27^ (6) The important aspect of the orientation of the injury differs between humans and animals (e.g., collapsed vertebrae damaging the ventral cord compared to dorsal contusions produced in animal models). In experimental settings, SCI models such as contusions are usually focused on the dorsal cord that affects sensory fibers as well as the CST and RST. In contrast, human lesions are heterogeneous in origin that often manifest in physical damage to the ventral spinal cord and are frequently combined with trauma to the brain and other organ systems.^28^,^29^

**Figure 1.**
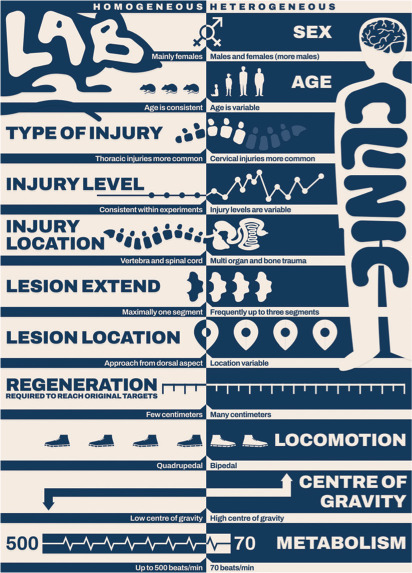
Differences between animal models of spinal cord injury and the clinical reality.

Differences in injury characteristics and comorbidities contribute to translational challenges. Nevertheless, despite differences between a rodent and a human, findings from rodent models can be very valuable, especially when results are interpreted while considering these anatomical and functional differences (**Figure 1**).

### Low preclinical variability challenges the translatability into clinical populations

SCI in humans is heterogeneous in nature. Injuries occur across the entire spinal column, with different severities, mechanisms, and demographics. Not surprisingly, it is difficult to capture this heterogeneity in animal models of SCI that aim to restrict variability by selectively controlling each of these variables to test a hypothesis. Most importantly, however, there is emerging preclinical evidence to suggest that changing a single demographic (sex, age, or even genotype) or injury variable (severity, mechanism, or location) can affect the outcome of experimental SCI and interventions. Some findings have identified differences in the magnitude of effect,^30^,^31^ and others have found opposing effects with one population experiencing a detriment and the other a benefit when a single variable was changed.^32^,^33^ With such examples slowly amassing in the preclinical literature, the inclusion of heterogeneity in animal models is quickly being appreciated as an imperative step toward improving the translatability of SCI interventions.^34^ Some caution is warranted, however, in that increased variability requires much larger animal numbers and more complex analyses, a challenge for the adoption of these practices.

Aside from preclinical research tending to restrict demographic and injury characteristics in animal models, many of the commonly used variables in preclinical design also deviate from the average clinical demographics. For example, we previously reported on the lack of heterogeneity in subject sex in rodent SCI models.^35^ In 2018, the vast majority (~70%) of National Institutes of Health (NIH)–funded rodent SCI research utilized only female animals.^35^ While the incidence of SCI is trending toward increases among females over time, the overutilization of female rodents is in stark contrast to the relative clinical incidence of SCI in males (~70%-80%) versus females (22%-27%).^36^,^37^ Further, a 2017 survey of animal models reported that the thoracic spinal level is the most common injury model studied (~80%)^38^ despite most SCI in humans occurring at the cervical spinal level (~60%-75%).^36^,^37^ And finally, younger animal models of SCI are the field standard, with most rodent models approximating an age analogous to a young human adult (16 years old to mid 20s as a best estimation). This is despite the average age at the time of SCI in clinical populations being middle-aged (~43 years old as of 2023),^34^,^39^ with a progressive increase of incidences in older adults resulting from falls.^40^,^37^,^41^,^42^ Ultimately each of these variables may interact with experimental manipulations to affect treatment responses.

There is a level of consistency in rodent models regarding the injury causes, which differs from the clinical population, where contusion-compression injuries are due to falls and motor vehicle accidents (~50%-80%)^36^,^43^ versus the frequency of contusion (~40%) or compression (~20%) SCI utilized in animal models.^38^ It is important to note, however, the distribution of injury biomechanics is not uniform amongst the population. For example, the frequency of falls increases with age in humans,^44^ which can often elicit more mild traumatic force and/or induce a pathology consistent with central cord syndrome. Both differently aged animals and central cord syndrome^45^,^46^ are rarely modeled in research.^47^ It is noteworthy to mention that nontraumatic forms of SCI such as degenerative myelopathy are also underrepresented in rodent research. Degenerative myelopathy causes direct damage to the spinal cord, often results in paralysis, and is the most common type of spinal dysfunction in adults.^48^

There are many reasons for the lack of variability in animal models and inconsistencies between clinical and experimental conditions. Male animals are difficult to care for after injury specifically regarding bladder care. Indeed, male mice experience greater weight loss and mortality relative to females under the same experimental conditions.^35^,^49^ High mortality is also likely a limiting factor when considering aged animals in SCI research.^34^ Animal care and concerns about morbidity and mortality also limit the use of cervical SCIs in basic science models. Animals with cervical SCI may lose respiratory function and experience high attrition rates (~25%-40%) after moderate-severe bilateral cervical contusion injuries.^50^,^51^,^52^ Milder bilateral injuries and unilateral injuries limit attrition after cervical SCI^53^,^54^,^55^,^56^,^57^ but present with anatomical and histological inconsistencies when compared to the human condition. Regardless of the reason, the lack of experimental and biological heterogeneity in SCI models has implications for the translational efficacy of therapeutic interventions. In the Gensel laboratory alone, we have observed (1) analgesics to treat SCI elicit sexually dimorphic effects,^58^ (2) the inclusion of compression in addition to contusion alters the inflammatory response to SCI,^59^ and age unpredictably alters the efficacy of SCI therapies.^34^,^60^ Regenerative therapies are likely to be affected in similar ways.^3^,^10^-^11^ Indeed, age reduces the regeneration, growth, and mitochondria functions of injured neurons.^32^,^61^,^62^,^63^ Injury biomechanics (e.g., hemisection or cut vs. contusion or crush) affect the extent to which axon regeneration versus sprouting from spared axons influences regenerative therapeutic outcomes.^1^ Axon regeneration responses vary depending on the proximity of injury to the cell soma,^64^ which has implications for upper motor neuron systems after cervical versus thoracic SCI. The spinal level of injury further influences regenerative interventions due to the relative ratio of gray and white matter at different spinal levels and the relative importance of spinal neurons to the pathophysiology.

Collectively, the heterogeneity of clinical SCI presents additional considerations regarding the applicability of regeneration therapies between commonly employed thoracic SCI versus less commonly modeled cervical SCI, as well as across different age ranges and injury types. While researchers can utilize relatively homogenous experimental paradigms to reduce variability and gain mechanistic insight into therapeutic manipulations, comparably controlled conditions are not available for clinical trials. The incidence rates for SCI are roughly 25 per 100,000 in North America and Europe, with even lower rates outside of military conflicts across the world.^43^ Therefore, any large-scale clinical trials are going to require multiple sites and are going to be extremely difficult to properly power under stringent inclusion/exclusion criteria. As a result, the translational application of regenerative therapies may include injury conditions not evaluated in preclinical work. We have recently advocated for including demographic variables such as age in preclinical testing stages to ensure no age-divergent effects exist that compromise clinical trial efforts.^32^,^34^ Considering the moderate effectiveness of treatments currently observed in animal models, this naturally contributes to the challenges with effective translation to the clinic.

## Overcoming Scientific Barriers to Regeneration

### Combinatorial treatments

Repairing the injured spinal cord to enable meaningful functional recovery will require combinatorial treatment approaches.^65^ Although many individual treatments have been advanced into clinical trials, the expected limited benefits likely impede their advance into clinical practice. There will likely never be a “silver bullet” that will solve the complex challenge and the multiple facets of SCI pathology. This is exemplified by the many obstacles an axon encounters during growth over long distances. First, neurons need to activate appropriate growth programs to facilitate a genetic regenerative response, which does not happen in most neurons of the central nervous system (or is not sustained).^66^,^67^ Second, axons will encounter various molecules (triggering different cellular messaging pathways) that impede the growth cone from advancing. Many inhibitory molecules have been well characterized in the literature including myelin-associated inhibitors in white matter and CSPGs in the perineuronal net surrounding cell bodies in the gray matter.^68^ Third, there is the issue of pathfinding and making connections to meaningful targets. With regard to regenerating damaged axons, or even to facilitate sprouting and structural plasticity from spared axons, we still do not know much about target selection of growing axons or how to facilitate appropriate or meaningful connectivity. Cystic fluid-filled cavities also form at the injury and complicate the ability to support axon growth and regeneration due to an absence of a growth substrate. Inevitably filling cystic cavities with a growth-permissive matrix will likely be required to support regeneration. Ultimately all the barriers to axon regeneration will need to be overcome with targeted therapeutic approaches that will require multiple strategic interventions.

The challenges with employing combinatory treatments are not to be underestimated. For example, interventions that address one mechanism might be disregarded prematurely due to a lack of efficacy in preclinical models or even in a clinical trial, despite potential benefits in a combination. Testing combinations often requires the addition of multiple control groups including different timing of their application. The addition of more control groups also increases the complexity of the study design, which requires more complicated statistical assessments that usually demand larger sample sizes in every group to appropriately power the experiments. Adding additional groups or procedures into a study further increases the potential for additional variability and lower confidence in the outcomes. Exploring combinational approaches is challenging, and consequently, progress is slow and cumbersome. However, given the recognition that combinatorial approaches will be essential to promote recovery, efforts are increasing to explore clinically available combinatorial approaches. In a recent review of clinicaltrials.gov, 28% of clinical trials included more than one intervention, and 75% of these included rehabilitation/training or exercise as one of the interventions. In preclinical studies, combinatorial approaches often include rehabilitation to facilitate meaningful rewiring/integration of new connections, which will be discussed in more detail below.^69^,^70^,^71^

## The Importance of Including Rehabilitative Strategies

### Preclinical use of rehabilitation

Rehabilitative training is the most frequently used and available approach to promote recovery (likely via plasticity) following SCI, and it is an essential part of clinical care. As such, one should consider training essential in preclinical research using animal models when testing pro-regenerative treatments on their journey to translation. Indeed, lessons from neurodevelopment and recent findings in animal models of SCI strongly suggest that rehabilitative training should be an integral part of preclinical animal testing.^72^,^73^ Studies in developmental neuroscience have described this process extensively, starting with the work of Hubel and Wiesel in the 1960s, where it was demonstrated that inactive neurons of the visual cortex fail to innervate the visual cortex resulting in a condition called cortical blindness.^74^ The phrase “use it or lose it” describes this phenomenon, which was demonstrated following primate models of cortical injuries by Nudo and colleagues.^75^ Spontaneous cage activity plays a big role in recovery,^76^,^77^ specifically following SCI. The group of D. Magnuson showed that activity is essential for harnessing injury-induced plasticity during spontaneous recovery of locomotor function. Animals restricted in their locomotor ability simply do not recover.^78^,^79^,^80^

Together these concepts suggest that treatments designed to promote axonal regeneration or neurite outgrowth might require rehabilitative training to facilitate meaningful rewiring of regenerating axons. Indeed, research by Garcia Alias and colleagues showed that the effect of chondroitinase ABC on recovery in rats with a cervical SCI depends on concomitant training.^78^ Similarly, our work has indicated that proinflammatory or neurotrophin treatments that increase plasticity effectively improve function in rats only when applied together with task-specific training.^69^ Although these findings have guided many animal studies to include rehabilitative training as part of their experimental design, there are still many questions to be answered. For example, how should training be applied to mirror the clinical scenarios? What is the intensity needed to make a difference (e.g., we have shown that high intensity is required to unmask beneficial effects).^81^ What are realistic points for subsequent training?

One should also consider the possibility that regenerative interventions could interfere with the stability of connections being made during rehabilitation. A destabilizing effect of a regenerative intervention might very well interfere with recovery induced from rehabilitation, or worse, facilitate aberrant connections that disrupt coordination and function. More research is needed to decide on appropriate/optimal and feasible timing of training when combined with plasticity promoting treatments. Inclusion of the exploration of appropriate intensity and timing of activity-based training will greatly add to the time, funds, and workload required to navigate the complex studies combining regenerative treatments with rehabilitation.

### Human rehabilitation

It is likely that, as with rodents, rehabilitative training is required to foster meaningful connections in humans with any axon regeneration-promoting intervention.^82^ However, there are many differences in rehabilitation conducted in animals and humans, and the cost and feasibility of rehabilitation in humans are challenging.^83^ Further, the optimal rehabilitation program for humans with SCI is unknown and likely varies per individual. Evidence in animals and humans supports high-intensity, high-dose, task-specific rehabilitation as a strategy to improve function after SCI.^84^,^85^ A recent study by Lotter et al. compared impairment-based approaches, including strength and balance training, to task-specific stepping and walking practice on walking outcomes in persons with motor incomplete chronic SCI over 20 sessions.^86^ The task-specific walking group demonstrated significantly greater increases in walking speeds relative to the impairment-trained group. This is consistent with clinical practice guidelines that recommend moderate- to high-intensity task-specific training for central nervous system dysfunction to improve walking speed and distance in chronic SCI.^87^ Work by Morrison et al. examined the locomotor effects of 120 high-dose, high-intensity training sessions consisting of manually assisted treadmill training, over-ground standing and stepping activities, and community integration tasks.^88^ For these individuals with chronic motor incomplete SCI, significant changes were noted in all walking assessments between baseline and study completion. Although it is easier to achieve high repetitions in walking activities versus upper extremity rehabilitation, the same concepts in lower extremity rehabilitation apply to the upper extremity.^89^,^90^ It is noteworthy that in humans rehabilitation studies are typically performed in chronic, incomplete SCI, with less recovery and response to rehabilitation noted with complete SCI.^91^

*Standard of care rehabilitation:* Despite evidence that supports high-intensity rehabilitation as a standard of care after SCI, constraints in the healthcare system challenge the ability to deliver optimal rehabilitation regimens. In the US Model Systems Centers, inpatient rehabilitation stays are short, averaging 12 days in acute care and 32 days in inpatient rehabilitation across all levels and severities of injury.^37^ In the United States, inpatient rehabilitation is required to be at least 3 hours/day. This time is divided between all rehabilitation disciplines, which may include group therapy sessions, resulting in a limited focus on recovery (vs. compensation). The majority of recovery-focused rehabilitation occurs during outpatient rehabilitation. However, the accessibility to outpatient rehabilitation is impacted by insurance, which limits outpatient rehabilitation to 20, 30, or at most 60 sessions.^88^ In order to achieve the high-intensity, high-dose rehabilitation likely required to promote change with regenerative approaches, a paradigm shift will be required.

*Rehabilitation in the context of clinical trials:* Due to cost constraints, many trials have no option but to use standard-of-care rehabilitation rather than high-intensity, high-dose, task-specific paradigms. Three of the major cell-based regeneration/remyelination trials conducted in North America (autologous activated macrophages,^92^ oligodendrocyte progenitor cells derived from human embryonic stem cells,^93^ and human central nervous system stem cells derived from fetal stem cells) did not incorporate rehabilitation beyond the standard of care.^94^ Two of these trials were in individuals with subacute SCI, where participants were in inpatient rehabilitation,^92^ whereas the third trial enrolled individuals 12 weeks or more following injury, so some of these individuals may have been in the outpatient setting.^95^

Several cell-based therapy trials (one in the United States and one outside the United States) incorporated standardized rehabilitation and in some cases high-intensity rehabilitation (olfactory mucosal autografts,^96^ intrathecal autologous bone marrow mesenchymal stem cell therapy,^97^ umbilical cord blood mononuclear cell transplant and Schwann cell transplants^98^) in studies recruiting individuals with chronic SCI. These were open-label, nonrandomized, trials where all individuals receive rehabilitation both pre- and post-intervention. Only one trial demonstrated improvement in a group with very high-intensity rehabilitation (6 hours/day, 6 days a week for 3-6 months); but given the study design, it is not possible to separate the effects of rehabilitation versus the cell-based intervention. To our knowledge, no completed regenerative clinical trial has included a treatment group, a treatment + rehabilitation group, and a rehabilitation-only group, which would be a substantial challenge for recruitment.

*Difference in animal vs. human rehabilitation:* In contrast to rodents with a severe contusion injury (analogous to a clinically “complete” SCI), persons with complete SCI have much less spontaneous functional recovery. Although there is emerging evidence that for individuals with motor complete SCI there is often some spared tissue that can allow neuromodulation approaches to facilitate motor and autonomic functions. Lower extremity rehabilitation in humans largely focuses on locomotor activities in people with motor incomplete SCI. Rodents participate in a lot of in-cage exploration and movement that contributes to recovery, whereas humans have far less spontaneous activity outside of rehabilitation, particularly in the inpatient rehabilitation phase. In terms of high-dose, task-specific rehabilitation, the number of repetitions in rodents appears much higher than in humans.^99^,^100^,^84^ A study 2017 in persons with incomplete tetraplegia undergoing rehabilitation in Canada found repetitions for upper extremity tasks totaling 115 over the entire course of inpatient rehabilitation (1-81 repetitions per session).^101^ This is in contrast to rats where repetitions might total 1600 (80 reaches, 5 days per week for 4 weeks).^81^

Although there is some evidence that the timing of rehabilitation matters when combined with regenerative approaches, we have no direct evidence in humans of the timing of rehabilitation when combined with a regenerative strategy. Most recovery in humans occurs within 6 months following SCI, with the steepest recovery curve in the first 3 months,^102^ however, there is also modest evidence of improvement in chronic incomplete SCI with intensive, high-dose, activity-based interventions.^103^ Identifying the optimal rehabilitation window when combined with a regenerative approach will be important if/when regenerative therapies are successful and clinically available.

In summary, evidence in humans and rats supports the use of high-intensity, task-specific rehabilitation to improve functional abilities after SCI. In rodents, there is evidence that rehabilitation improves the effects of a regenerative approach and that timing matters. However, in humans, high-intensity rehabilitation is not the standard of care, and it is difficult to achieve and financially support the number of repetitions and intensity required, a challenge that needs to be considered when translating any regenerative intervention.

### The Role of Gray Matter

In the nineties, functional deficits after spinal cord injury were frequently considered to be based on white matter injury,^104^,^105^,^106^ and spared white matter was a common metric for the size of a spinal lesion.^104^ Based on this line of thinking and the animal ethic and animal care advantages (relative to cervical injury), thoracic injuries were the most common injury model in rodents. With the introduction of a standardized contusion model and associated validated functional outcome measures such as the BBB locomotor score, the use of thoracic contusions became the standard in the field.^107^,^108^,^109^ Even the annual summer school at The Ohio State University was originally dedicated to teaching students the injury model and associated outcome measures. This model was preferred to cervical injuries, especially contusions, mainly due to animal care considerations. Furthermore, SCI was considered a white matter problem. Damage to enlargements was not considered that important. However, the focus on white matter damage can create problems; for example, in various unpublished experiments, we have experienced that treatments, even as “straight forward” as rehabilitative training, fail to repeat efficacy in promoting recovery when performed in contusion models as compared to cut lesions. A lack of reproducibility potentially can be explained by cut and hemi- or dorsal-section lesion injuries in rats being performed with small blades and extending only ~100 μm, whereas contusions are performed with impactor tips of 1 to 1.5 mm. The associated gray matter damage varies greatly; although this does not make a big difference in thoracic lesions, the loss of motoneurons and interneurons at the cervical level creates a bottleneck for recovery. Even if injured neurons may/did regenerate or at least show collateral growth, they are deprived of targets and the essential circuitry of paw/hand and arm use are affected significantly. Considering that motoneurons and their associated interneuron circuitry can spread over various segments, the impact of a contusion spreading over about one segment likely can be buffered by the remaining motoneurons, as a muscle can retain its output up to a loss of 80% of its innervation due to plasticity at the neuromuscular junction. This bottleneck for recovery likely reaches a critical point in humans, where cervical lesions are frequently spread over more than two segments, limiting the ability of any axonal growth to affect recovery. Targets for regenerating neurons are simply missing and muscles lose their input neurons. Additional treatments for these lesions will be required, including cell replacement using transplanted stem cells for example.

## Stem Cell Therapies as a Leading Translational Effort for SCI Regeneration

### Evidence from preclinical literature to support translation

One of the more exciting and debatably overpromised therapeutic advances for the SCI field has been the use of stem cells as regenerative treatments. Despite initial enthusiasm and significant advances in cell therapies for SCI in the past 20 years, there are still no cell therapies approved by the US Food and Drug Administration (FDA) for use to treat SCI. Although many transplantation-based clinical trials have emerged,^95^ we do not yet fully understand the realistic potential for cell therapies to restore function after SCI, thus we do not yet know the upper and lower limit of their therapeutic potential.

*What evidence exists to support that axon regeneration or neuronal relays contribute to functional recovery after neural stem cell transplantation?*

A comprehensive list of the various types of cells used for transplantation in preclinical models and proposed therapeutic mechanisms can be found in prior literature.^110^ However, we must first classify cells as either those capable of replacing central nervous system architecture or those that are not. Neural stem cells (NSCs) can function to replace damaged neurons, myelin, and astrocytes, whereas most other cells used for transplantation do not. There is one exception, Schwann cells, that also may be capable of remyelinating damaged axons.^111^ Decades of preclinical literature identified that injecting cells of most kinds into the nervous system can exert therapeutic effects through a secretome that is rich in trophic factors and growth factors or other anti-inflammatory cytokines, which can aid in the regeneration of damaged axons, facilitate plasticity of local circuitry, and promote survival from secondary injury. Even NSCs, which are utilized primarily for their potential to replace lost nervous system tissue, secrete pro-growth/survival factors.^112^ The very nature of cell grafting can therefore elicit a multitude of effects that can improve outcomes after SCI.

To date, the evidence to suggest that cell transplantations can improve neuronal survival, regeneration, and plasticity in animals is strong.^113^ However, the extent that regenerating axons, or the formation of neural relays from transplanted neurons, convey meaningful improvements in function is still limited and debated.^114^ Even though evidence that neural synapses can form between endogenous axons and transplanted neurons does exist,^114^,^115^ evidence that neuronal relays can improve function meaningfully remains limited and understudied. Potential approaches should consider combinations of approaches including histology (e.g., tract tracing and synaptic markers, electrophysiology, and potentially chemo- or optogenetics), which is feasible in animal models but not humans. Work to prove mechanistically that transplanted neurons form meaningful functional relays was attempted in prior reports.^115^,^116^

In work by Abematsu and colleagues, mouse NSCs containing a humanized diphtheria toxin receptor were transplanted and were found to improve motor functions, differentiate into neurons, and make synaptic connections with host tissue.^116^ The NSCs were subsequently ablated by administering diphtheria toxin after functional improvement were gained. Consequently, the functional abilities returned to untreated levels after the ablation of transplanted NSCs. While this experiment may provide some evidence that the survival of the graft is required to sustain functional improvements from NSC transplantation, there are some caveats that limit the conclusion as to whether the transplanted cells formed meaningful functional neuronal architecture. First, growth factors and trophic factors secreted from transplants can modulate endogenous synaptic functions. Thereby ablating a transplanted graft may abolish the supportive secretome that could be sustaining the observed improvements. Next, it could also be possible that ablating the graft using diphtheria toxin exacerbated inflammation leading to further damage of the spinal cord. Finally, the authors report that only 20% of the surviving cells differentiated into neurons. Therefore, other cell types like oligodendrocytes could have remyelinated damaged axons and induced much of the benefits from the graft. Historic evidence has found that the benefits from NSC transplantations are largely derived from oligodendrocyte differentiation and the formation of new myelin, rather than other mechanisms of action.^117^,^118^ While the work by Abematsu and colleagues was of excellent design, reflection on possible alternative explanations for the observed outcomes highlights the challenges of proving the contribution of neural relays to functional improvements.^116^

A more recent report similarly attempted to elucidate the contribution of neural relays on functional outcomes after NSC transplantation but used inhibitory designer receptors that are exclusively activated by designer drugs (DREADD) to silence neuronal signaling from the graft.^119^ Neuronal silencing using DREADDs arguably would not eliminate much of the graft-derived secretome or induce inflammation from ablating cells.^119^ Kitagawa and colleagues observed improvements in locomotor abilities after grafting NSCs in mice and subsequently detected a small but significant decrease in locomotor abilities while silencing the transplanted neurons with DREADDs. Although a small amount of function was lost during neural silencing, most of the functional improvements persisted.^119^ These findings further support that much of the therapeutic properties derived from NSC transplantations is not related to neural relay.^117^,^118^ They do however, provide some evidence that locomotor improvements can be supported in part by the electrophysiological properties of the graft. Collective evidence supports the role of grafting NSCs as a viable means to improve outcomes after SCI. These outcomes could be elicited by several possible mechanisms. Neural architecture can be supported and improved through remyelination derived from transplanted oligodendrocytes. However, the contribution of neural relays to observed locomotor improvements remains less clear. It also remains possible that shorter distance connections made from transplantations in cervical models may be capable of restoring larger amounts of function to upper extremities or respiratory centers when axon projections are not required to grow extended distances down the spinal cord.

### Moving cell transplantations into clinical trials: Demonstrating safety and efficacy versus understanding the mechanisms of improvement

There is an urgent need to expedite safe and effective interventions forward into humans who are suffering with SCI.

Recently, FDA-regulated trials of stem^93^,^120^,^121^,^122^ and Schwann cell^123^,^124^ transplants have been completed. Approaches have conferred a reasonable safety profile with up to 10 years follow-up in one case, supporting further exploration. As of April 2023, there were 52 SCI transplantation studies registered on clinicaltrials.gov (search term “spinal cord injury” for disease/condition and “cell transplantation” for other), with many more being conducted throughout around the world. Yet with regard to cell transplantation approaches and a 60- to 70-year history for use in preclinical models of SCI,^125^ we still do not fully understand the mechanisms by which improvements are gained. Moreover, with regard to strategies aimed at neuron replacement, we do not yet know the best methods to achieve replacement of neural architecture or the realistic potential for neuronal relays to meaningfully integrate into the spinal cord. Advancing cell transplantation into clinical trials despite our limited understanding of mechanisms and best approaches highlights the contrast between a need for clinical translation and a need for more basic/preclinical research.

With regard to neuron transplantation, the limitations in our knowledge of how to best obtain a desired cell-replacement outcome are apparent and merit more development in basic/preclinical research. With such limitations in our understanding, can we yet answer what are the upper limits to the therapeutic potential of cell transplantations? Although much of our discussion thus far has focused on neuron replacement strategies, to date most of the registered clinical trials for stem cell-based therapies utilize nonneural-based cell transplantations. Of the 52 registered clinical trials, only eight have proposed to use NSCs or neural progenitor cells (NPC) directly, and 45 are utilizing nonneural cells, with one of these studies proposing to use both as independent arms when seeded on scaffolds.

### Interpreting results from clinical trials matters when design deviates from preclinical strategies

Recent human clinical trials reveal interesting findings. First, it should be noted that there is a major contrast between how stem cell therapies have been used in humans and animal models. The majority of cell transplantation studies in animal models have focused on transplantations early after injury, while delayed transplantations have not been performed nearly as frequently. When chronic animal studies (which are ill-defined and range from 4 weeks to many months) are performed, most experiments report no effect or significantly lower effects compared to acute/subacute experiment counterparts, particularly with the use of NSCs/NPCs.^126^,^127^,^128^ Yet, despite the preclinical literature overwhelmingly performing early transplantation approaches,^128^ clinical trials have a much higher representation of chronic transplantations.^129^ Of the at least 52 registered clinical trials involving stem cell transplantations (search terms on clinicaltrials.gov were “transplantation” for the condition of “spinal cord injury”), 25 registered studies were designated for chronic state SCI alone (here defined around 6 months post injury), with 15 more studies including arms to study both acute/subacute and chronic stages of injury (40/52 trials are investigating transplantations in chronic SCI). Further, preclinical transplantation studies tend to use anatomically incomplete SCI models,^34^ whereas 21 of the 52 registered clinical trials include only motor and sensory complete SCI (American Spinal Injury Association [ASIA] Impairment Scale - AIS A classification), and only two registered trials are designated for only incomplete (AIS B-D) SCI. Although preclinical data to support the use of cell transplantations in both severe/complete and chronic stages of SCI does exist, the evidence for efficacy is far less compared to incomplete and subacute transplantation strategies.^128^,^130^

It is important to note that clinical trial design benefits both practically and scientifically from the use of chronic and complete conditions of SCI. For one, there are safety concerns to be considered when injecting a bolus of biological mass into the spinal cord^131^,^132^ with the potential to cause physical trauma and potentially worsen outcomes. For this reason, thoracic, motor, and sensory complete SCI is often preferred under the reasoning that any potential damage will have less impact on function relative to incomplete or cervical SCI. Next, use of chronic conditions of SCI offers a more realistic outlook on an individual's ability to function as further spontaneous recovery is limited, adding further confidence in the safety of the transplantation. From an experimental design standpoint, the extent of spontaneous recovery in acute/subacute stages of injury significantly challenge the ability to interpret the outcomes of early interventions using anything, stem cells included. Use of chronic injury conditions allows for the establishment of an individual's own baseline, thereby enhancing the confidence in detecting small effects. There are advantages to using chronic complete conditions of SCI for cell transplantation clinical studies. However, it is important to consider whether the mechanism makes sense and the preclinical evidence supports testing in chronic SCI. If clinical study design deviates so substantially from critical elements of preclinical research, how can we begin to compare or conclude evidence of efficacy between preclinical and clinical findings?

There are potential confounds for interpreting results from both acute/subacute and chronic transplantation experiments in humans. For acute/subacute transplantation experiments, it is difficult to separate observations of recovery caused by sporadic recovery from recovery caused by the graft. Many individuals recover some function in the months following SCI without intervention, albeit the extent of functional return remains highly variable.^37^,^38^,^102^,^133^ Improvements in function can even be detected through rehabilitation alone, even in chronic SCI, where the transplant could induce inflammation and potentially drive plasticity during rehabilitation training.^70^ Rehabilitation as a potential effect modifier challenges the ability to separate improvements gained from rehabilitative training from the effects of the cell graft.^103^,^134^,^135^ This confounding variable has been recognized and is partially being integrated into the design of future studies. For example, a control group of participants in the StemCyte clinical trial (ClinicalTrials.gov ID: NCT03979742) is being put through a rigorous rehabilitation regime without receiving stem cells to control for an effect of rehabilitation.

Currently, our emerging evidence for stem cell transplants into humans supports a reasonable safety profile, worthy of further exploration for efficacy. With the risks associated with spinal injections and immunosuppression procedures, one needs to consider the risk-benefit ratio. Again, these considerations and decisions should not be made without the perspectives of individuals living with SCI.

### Adverse events and caveats with immune suppression

As noted, cell transplantations are not free from risks, such as the potential for mechanical damage associated with intraparenchymal injection. Further, one of the biggest concerns with the use of cell transplantation approaches is the threat of tumor formation. To date, no tumors have been found in any clinical trial,^136^ but uncontrolled growth of transplanted stem cells has been observed periodically in preclinical literature.^137^,^138^,^139^,^140^ The circumstances underlying why and when cell transplantations may undergo uncontrolled growth have not been examined thoroughly, but the risks for tumor formation may be increased with combinational approaches. For example, Stewart and colleagues observed tumor masses forming when NSCs and mesenchymal stem cells (MSCs) were co-transplanted but were not observed when either cell was transplanted alone.^138^ May and colleagues observed uncontrolled growth of Schwann cell grafts when seeded in hyaluronan-methylcellulose or laminin and fibronectin hydrogels^137^ in animal models.

While observing that tumor growth is rarely reported, there is a need to identify mechanisms that predict such adverse outcomes in order to increase the safety and feasibility for clinical translation. The risks of uncontrolled growth or tumor formations are especially highlighted with the use of undifferentiated pluripotent stem cells such as embryonic (ESC) or induced pluripotent stem cells (iPSCs).^141^ It will be important to ensure proper differentiation and lineage commitment of iPSC cell lines prior to transplantation in order to avoid the risks associated with tumor growth from undifferentiated cells. Currently, a long-term follow-up from iPSC-derived oligodendrocyte progenitor cell transplantations into humans has not found any evidence for overgrowth of the transplant in enrolled participants.^93^

Notably, the transplantation of non-autologous cells requires the use of immune suppression paradigms to restrict rejection of the graft. We have learned through clinical utility of methylprednisolone that suppressing the immune system shortly after SCI can increase the prevalence and severity of some adverse events.^142^,^143^,^144^,^145^ Use of methylprednisolone acutely after SCI increases the odds of gastrointestinal bleeding, lung infection/pneumonia, and severe infections as well as sepsis.^142^,^143^,^144^,^145^ Although not observed in various clinical trials, it is therefore still possible that similar effects could occur if immunosuppression is delivered acutely after SCI to support stem cell grafts. However, studies transplanting allogenic stem cells have reported favorable tolerability of immune suppressive procedures.^93^,^146^ Further, because most registered clinical trials are using autogenic transplants of MSCs, bone marrow mesenchymal stroma cells (BMSCs), or Schwann cells, immune suppression has not often been required.

Current reports on adverse outcomes associated with immune suppression are difficult to interpret due to the uncertainty with comparing the frequency of adverse events after cell transplantation to those deri ved from other unaffiliated causes. For example, urinary tract infections are common amongst individuals living with SCI, but they are still documented as adverse events following cell transplantations. It is foreseeable that immuno-suppression could increase the likelihood of developing urinary tract infections or other infections related to pneumonia or pressure ulcers. However, several clinical trials have reported no serious adverse events related either to immunosuppressive procedures or to the cell transplantations,^93^,^146^ with some moderate adverse events infrequently observed (infection treatable with antibiotics^120^). Several less severe adverse events known to be caused by immunosuppression are, however, commonly reported, such as nausea, low magnesium levels, and urinary tract infections.^93^,^146^

Yet another deviation between preclinical and clinical research methods exists in immuno suppression approaches. Today, many research protocols use immunodeficient animal models for cell transplantation studies, or use syngeneic transplantations that make use of inbred strains of rats such as Fisher's that have less rejection of cells obtained from the same strain.^147^,^148^,^149^ However, dating back to prior use of animal models, pharmacological immune suppression was essential to the survival of grafted cells coming from allogenic or xenogeneic sources.^149^ In preclinical studies utilizing immune-suppression paradigms, rats and mice are sustained on immunosuppressants for the duration of the study. Recent clinical trials, however, are utilizing different immunosuppressant paradigms. Specifically, when immunosuppression is used, it is common to observe immunosuppressants delivered for only 9 to 15 weeks post-transplantation.^93^,^146^ Overall, even if allogenic transplantation approaches are used, the use of immunosuppressants is reported to be well tolerated, and adverse events related to immunosuppression have been minimal and expected.

### Does the current evidence of efficacy with cell transplantation approaches merit the use in people?

There is a growing consensus in both preclinical and clinical literature that supports the safety profile for cell transplantation procedures into SCI, however evidence of efficacy, while present in animal models, should be further investigated in clinical populations. We are left with some imperative questions with regard to a path forward. What level of efficacy and reproducibility are we willing to accept as sufficient to make cell transplantations available as a standard of care? More importantly, who should make the ultimate decision about “is it worth it”? There is no question that we still need to identify the conditions that provide the strongest evidence of efficacy for cell transplantations, that is, cell source, timing, location, injury severity and characteristics, outcomes affected, etc. However, qualifying the value that an intervention may have is ultimately a decision best made by the individuals receiving the intervention. From one point of view, what we as scientists or clinicians may deem as an effect too small to be meaningful, could be a life-changing influence to someone living with SCI. However, from a different perspective, observing a statistically significant improvement in an outcome may not affect quality of life (which is, by the way, not assessed in animal models). For example, if the outcome of a treatment delivered to cervical SCI could improve finger dexterity or force even a small amount, the effects could be life changing to those living with disrupted hand movements. However, a small incremental gain to leg movement may not change the dependence on a wheelchair or affect day-to-day functions enough to merit the risks of the procedure. In contrast, a small gain in bowel or bladder or sexual function would be immensely meaningful.^150^ Both of these hypothetical examples highlight a need to both (1) expand analysis of potential benefits to include more systems affected by SCI such as bowel and bladder control, sexual dysfunction, autonomic dysreflexia, or pain (consider SCI as a multi-system disorder) and (2) include the opinions and appraisals of advocates living with SCI in the decision-making process during the analysis of research outcomes, study design, and future policies involving the use and risk-determination of cell-based therapies.

### Limited Transparency and Replication in Research

A challenge in research that goes beyond the field of neuroregeneration is the manner in which results are disseminated. The current approach has created a dramatic publication bias where mainly so-called positive results are published. When treatment strategies are explored, it is generally more difficult and less rewarding to publish findings where an intervention had no beneficial effect. Consequently, it is a more productive approach for a researcher not to spend the time analyzing results and or performing additional controls in order to be potentially able to publish the results in a lower-tier journal. This results in a huge amount of “dark data” that is untapped, representing billions of dollars in funding. The resulting bias can give the perception that interventions in animal models are tested regularly and that they are promoting regeneration, repair, and recovery reliably. Therefore, when clinical trials potentially fail, the blame is quickly put on inappropriate animal models. A similar unrewarding venture for researchers is replication studies. There is a strong culture toward “new results,” and there are many barriers to fund and publish study replications. Although agreed upon to be of high importance to move a treatment forward, such studies require a lot of work that potentially will not be rewarded with a publication and certainly do not score well for innovation when applying for funding. Yet another problem is not publishing the necessary details to comprehend studies in humans and animals alike (this can go as far as not mentioning the sex of the subjects in animal studies). Frequently standards have been suggested (e.g., arriveguidelines.org), but they are not generally followed. Many manuscripts do not describe the type of experimental kits for biomarkers, detection ranges, noninjured and SCI range values, and clinically significant changes; this limits the ability to analyze and compare results and to replicate studies. Lastly, one must accept that many *in vivo* experiments are performed with relatively low power, suffering from little inherent variability, and thus tend to produce false negative and positive results. As mentioned previously, the false positives get published and will rarely be repeated; as a result, the literature presents itself as a distorted and biased reality. On a positive note, this problem has been generally recognized, and the research climate is undergoing a dramatic change, with data management and sharing mandates from the majority of funding bodies. Other ideas to tackle these challenges include repetition of studies, platforms trials, and contract research organizations. Overall the balance between explanatory research (to create hypothesis and treatment avenues) versus confirmatory research to carefully test these hypothesis needs to be reevaluated.^151^

### The Future

Where should we go from here? There are encouraging changes occurring in the field, and there are obvious immediate actions to promote regenerative research and the translation to people living with SCI. Our recommendations include the following:

- Consider study designs, outcome measures, and populations that reflect clinical reality. For example, we need to identify and then match clinical populations to the preclinical research and use data to guide clinical trial design.
- Engage in realistic and transparent reporting without hype and overinterpretation of findings, reporting standards, and publication of all data (being negative or positive) in repositories such as the Open Data Commons for Spinal Cord Injury (ODC-SCI.org).^152^
- Recognize that personalized approaches may be required in SCI (e.g., different lesions require different interventions), particularly with emerging preclinical evidence to suggest that interventions may not behave the same way in different demographic populations or injury characteristics.
- Include the perspective at all levels of research, including and understanding the risk/benefit ratio of treatments.
- Change the culture of funding and publishing only novel research and the role of high-impact publications to foster career development. Experimental treatments can be properly advanced in the translational pipeline only when funding and recognition for translational research goes beyond the usual explorative and screening process. For example, investigators evaluating promising interventions should be funded for independent evaluation and testing in clinically relevant models; in some cases, additional mechanistic interrogation (fund advancing knowledge of therapeutic approaches in addition to identifying actionable targets and elucidating mechanisms) may not be necessary.

In conclusion, there are many challenges in translating regenerative treatments from the bench to the bedside. Many of these problems are deeply rooted in the academic culture and not necessarily limited by shortcomings of animal models. This raises hope for many ongoing efforts to facilitate the process of finding regenerative treatments for spinal cord injury.
